# Methane is an important selection factor in microbial community assembly along the Greenland Ice Sheet margin

**DOI:** 10.1128/aem.00875-26

**Published:** 2026-09-15

**Authors:** Lia C. P. Wentzel, Petra Klímová, Tyler J. Kohler, Jade E. Hatton, Anna Stehrer-Polášková, Jakub D. Žárský, Jakub Trubač, Philip A. Píka, Jon R. Hawkings, Eva L. Doting, Jack G. Murphy, Marek Stibal

**Affiliations:** 1 Department of Ecology, Faculty of Science, Charles University 37740, Prague, Czechia; 2 Environment Centre Wales, UK Centre for Ecology & Hydrology 41865 , Bangor, United Kingdom; 3 Institute of Geochemistry, Mineralogy and Mineral Resources, Faculty of Science, Charles University 37740, Prague, Czechia; 4 Geosciences, Environment and Society Department, Université Libre de Bruxelles 26659, Brussels, Belgium; 5 iC3—Centre for Ice, Cryosphere, Carbon and Climate, Institute for Geosciences, UiT – The Arctic University of Norway, Tromsø, Norway; 6 Department of Earth and Environmental Science, University of Pennsylvania 6572, Philadelphia, Pennsylvania, USA; 7 Department of Arctic Ecology, Norwegian Institute for Nature Research 8019, Tromsø, Norway; Colorado School of Mines, Golden, Colorado, USA

**Keywords:** microbial community assembly, Greenland Ice Sheet, methane, climate change, 16S rRNA amplicon

## Abstract

Ice sheet beds host metabolically active microbial communities and serve as sites of methane (CH_4_) production, consumption, and mineralization. Microbially derived CH_4_ is released from the western margin of the Greenland Ice Sheet (GrIS) as meltwater-dissolved CH_4_ (CH_4(aq)_) at concentrations spanning five orders of magnitude (from ~0.4 nM to ~50,000 nM), indicating a strong gradient in its availability. Here, we test the significance of CH_4_ as a determinant of microbial assemblage structure along the GrIS’s western margin by serving as an important carbon and energy source. We sampled microbial assemblages from glacial runoff spanning a ~2,000-km transect and characterized them using 16S rRNA amplicon sequencing. The assemblages were dominated by the genera *Rhodoferax*, *Polaromonas*, *Methylotenera*, and *Crenothrix*, the latter an important methanotrophic group. Distance-based redundancy analysis identified CH_4(aq)_ concentration as the strongest predictor of microbial assemblage structure. Differential abundance analysis revealed a coupling between organisms involved in complex organic matter degradation and CH_4_ cycling, suggesting microbial interactions linking methanogenic and methanotrophic processes. Consistent with these patterns, phylogenetic bin-based null modeling indicated CH_4_ is a key driver of community assembly, with higher CH_4(aq)_ concentration sites (i.e., >4.9 nM) exhibiting a stronger signal of homogeneous selection, whereas sites below this threshold were more strongly influenced by stochastic processes, particularly drift and dispersal limitation. Together, these results suggest that CH_4_ exerts a strong deterministic influence on microbial assembly at the GrIS margins and highlight the role of these ecosystems in carbon cycling and CH_4_ dynamics in a warming Arctic.

**IMPORTANCE:**

Our findings provide new insight into the ecological role of CH_4_ in the Greenland Ice Sheet ecosystem by linking its concentration gradient to the microbial community assembly process at the ice sheet margin and contribute to understanding carbon cycling in this rapidly warming ecosystem.

## INTRODUCTION

Microbial communities inhabiting glacier and ice sheet beds and margins remain underexplored due to the logistical challenges surrounding direct access to these habitats. Consequently, previous studies have been spatially restricted and primarily relied upon indirect observations, including analyses of cells entrained in exported meltwater during the melt season (1–3). While some studies have characterized the taxonomic composition and inferred metabolic potential of these exported assemblages (3–7), the ecological processes and selective forces that drive microbial community assembly remain unclear.

Methane (CH_4_) emissions have been detected at glacier and ice sheet margins worldwide, linked to meltwater drainage pathways and discharge (8–11). Along the western margin of the Greenland Ice Sheet (GrIS), this exported CH_4_ is of microbial origin and produced predominantly via acetoclastic and possibly also hydrogenotrophic methanogenesis in subglacial sediments (9, 12, 13). The concentration of dissolved CH_4_ (CH_4(aq)_) in the GrIS meltwater varies in time and space, spanning five orders of magnitude, from below atmospheric equilibrium (<4.9 nM) to ~50,000 nM (13). This wide range suggests that meltwater CH_4(aq)_ concentrations are dynamic and reflect underlying biogeochemical and hydrological processes. Given that CH_4(aq)_ represents both an end product (methanogenesis) and a substrate (methanotrophy), its spatial variability along the western GrIS margin may indicate distinct subglacial and/or ice-marginal redox niches and metabolic regimes. Such biogeochemical gradients have the potential to impose strong environmental filtering, favoring specialized microbial guilds and promoting deterministic community assembly.

Here, we test the hypothesis that CH_4_ acts as a significant environmental filter and selection factor for microbial community assembly at the margin of the GrIS. We collected meltwater samples from 16 locations along a ~2,000 km north-south transect across the western margin of the GrIS, analyzed their microbial assemblage structure, and compared the community data with *in situ* measurements of CH_4(aq)_ concentration and other meltwater characteristics. Using a combination of multivariate statistics and phylogenetic null modeling, we assess the influence of CH_4_ concentration on microbial community assembly relative to other environmental variables and determine the relative importance of deterministic (environmental selection) versus stochastic (ecological drift, dispersal) assembly processes.

## MATERIALS AND METHODS

### Field sites and sampling

**Fig 1:**
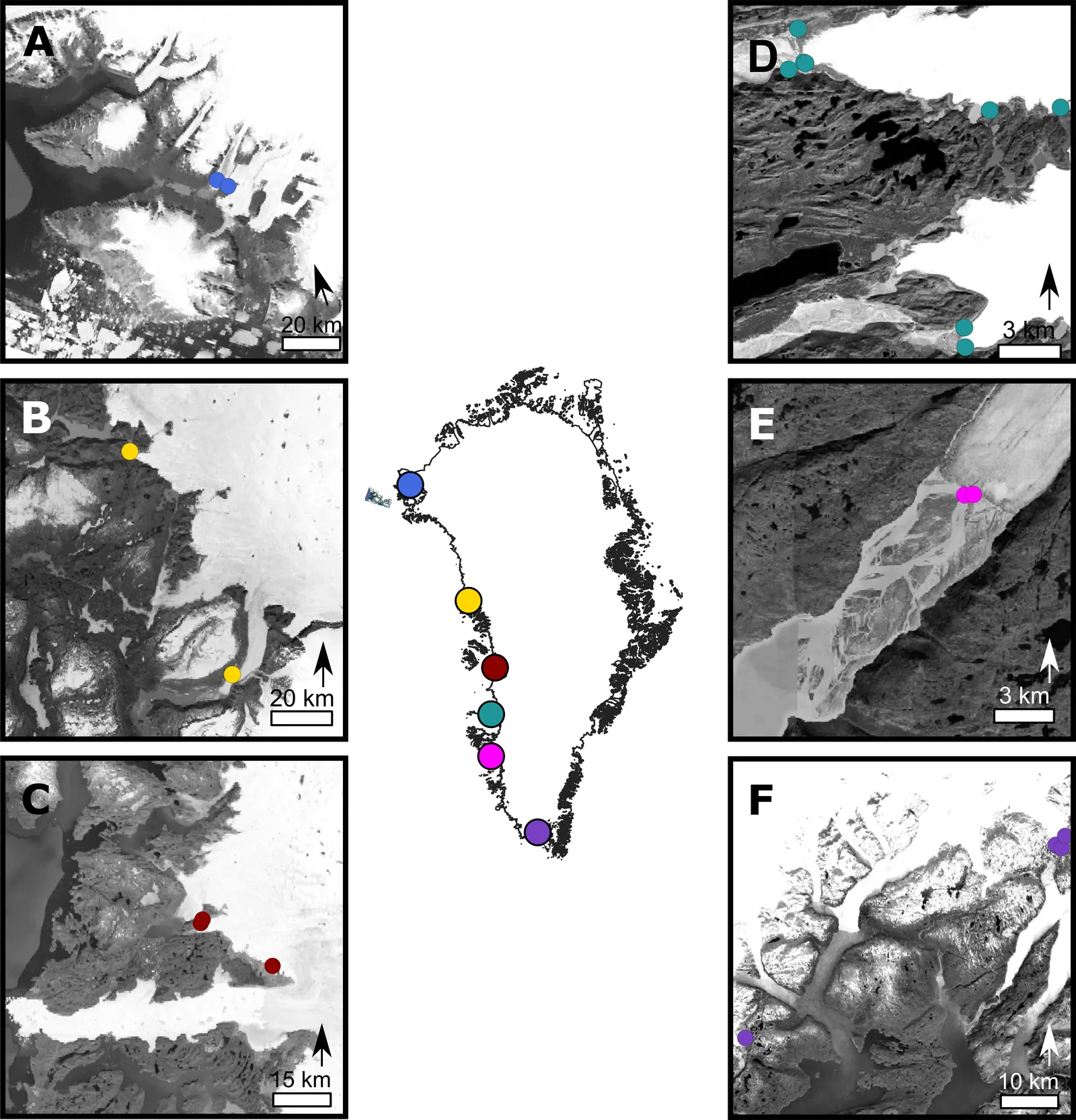
Map showing the six regions sampled along the western margin of the Greenland Ice Sheet, with inset panels providing the precise location of the 16 sampling sites. Listed counterclockwise from the upper left: (**A**) Qaanaaq, (**B**) Upernavik, (**C**) Ilulissat, (**D**) Kangerlussuaq, (**E**) Nuuk, and (**F**) Narsarsuaq. Background images were sourced from Google Earth, using the data set from the U.S. Geological Survey and the International Bathymetric Chart of the Arctic Ocean (IBCAO), and compiled using QGIS.

Samples were collected along the western margin of the GrIS, forming a latitudinal transect from Qaanaaq to Narsarsuaq (Fig. 1). Sixteen meltwater streams draining the GrIS were sampled over three summer melt seasons (August–September 2021, July–September 2022, and July 2023, with some rivers revisited across multiple years; Table 1). We collected a total of 26 site-date samples (i.e., individual sampling events). Each sample was processed in triplicate or quadruplicate (except one), resulting in a total of 78 samples for microbial analyses (Table 1; Table S1).

**TABLE 1:** Characteristics of collected samples^*a*^

Region	Sample	Date sampled	Latitude	Longitude	No. of replicates	CH_4(aq)_ range (nM)
Qaanaaq	NW1	15/08/2021	77.719667°	−69.019667°	3	≤4.9 (Atmospheric equilibrium)
Qaanaaq	NW2	14/08/2021	77.71525°	−68.91677°	3	4.9–20
Upernavik	NW3	22/07/2022	72.65713°	−54.0292°	3	80–490
Upernavik	NW4	20/07/2022	72.35803°	−53.5122°	3	40–80
Ilulissat	CW4-a	31/08/2021	69.329768°	−50.244147°	4	20–40
Ilulissat	CW4-b	15/07/2023	69.33613°	−50.23464°	3	20–40
Ilulissat	CW5	29/08/2021	69.249883°	−49.879465°	3	40–80
Kangerlussuaq	SW3-f	09/09/2021	67.195352°	−50.342082°	3	40–80
Kangerlussuaq	SW3-a*	06/09/2021	67.180635°	−50.336008°	3	NA
Kangerlussuaq	SW3-b	10/07/2022	67.1803°	−50.3343°	3	80–490
Kangerlussuaq	SW3-c	09/07/2022	67.17771°	−50.3533°	3	80–490
Kangerlussuaq	SW3-d	15/08/2022	67.1803°	−50.3343°	3	4.9–20
Kangerlussuaq	SW4	04/09/2021	67.159°	−50.129306°	4	≥490 (High-CH_4_ site)
Kangerlussuaq	SW5-a	05/08/2021	67.15968°	−50.0503°	3	≥490 (High-CH_4_ site)
Kangerlussuaq	SW5-c	03/09/2021	67.15968°	−50.0503°	3	≥490 (High-CH_4_ site)
Kangerlussuaq	SW5-d	13/07/2022	67.15968°	−50.0503°	3	≥490 (High-CH_4_ site)
Kangerlussuaq	SW6-a	11/09/2021	67.064807°	−50.161167°	3	40–80
Kangerlussuaq	SW6-b	09/07/2023	67.0648°	−50.1609°	3	80–490
Kangerlussuaq	SW7	10/07/2023	67.05626°	−50.16009°	3	≥490 (High-CH_4_ site)
Nuuk	SW12	31/07/2022	65.19899°	−50.6738°	3	20–40
Nuuk	SW12-b*	01/08/2022	65.19912°	−50.6684°	3	NA
Narsarsuaq	SW13	04/08/2022	61.14541°	−46.1282°	3	4.9–20
Narsarsuaq	SW14-a	07/08/2022	61.4273°	−45.13174°	3	4.9–20
Narsarsuaq	SW14-b	08/08/2022	61.4286°	−45.13449°	3	20–40
Narsarsuaq	SW15	08/08/2022	61.42386°	−45.11658°	3	≤4.9 (Atmospheric equilibrium)
Narsarsuaq	SW15-b	08/08/2022	61.44128°	−45.1042°	1	≤4.9 (Atmospheric equilibrium)

^*a*^ Samples marked with an asterisk (*) were not used in any of the analyses because of an insufficient number of reads and/or low quality.

To minimize potential proglacial influence, we sampled meltwater as close to the ice margins as possible, either at an upwelling (proglacial spring) or a subglacial portal (13). At each sampling event, *in situ* measurements of temperature, pH, conductivity, and dissolved oxygen were obtained using a handheld multi-sensor meter (Multi 3430, WTW). Water for microbial analyses was collected in acid-washed (2N HCl) 1-L HDPE Nalgene bottles after triple rinsing with sample water. Samples were filtered using 20-mL clean (2 N HCl), sample-rinsed syringes through Sterivex Filter Units (0.22 µm) until they clogged (150–500 mL). Filters were flushed with air, capped, and kept cool (~5°C) and in the dark until frozen (at −20 °C), typically within 5 hours (2–3 days for samples NW1, NW2, NW3, NW4, CW4-a, and CW5) of collection before DNA extraction.

The Sterivex-filtered glacier meltwater was collected into pre-cleaned (2 N HCl) HDPE bottles for major ions and nutrient analysis, or furnaced glass vials with PTFE-lined caps for dissolved organic carbon (DOC) analysis. Collection bottles were rinsed three times with filtrate prior to final sample collection. Samples intended for DOC analyses were collected last (after >150 mL passed through filters) and acidified immediately with 160 µl of 6 N HCl. Water chemistry samples were kept cool and in the dark until refrigerated (DOC, major ions) or frozen (nutrients). DOC was measured with an enviroTOC (Elementar) using high-temperature catalytic combustion with infrared detection after sparging with ultrapure air to remove dissolved inorganic carbon species. Soluble reactive phosphorus (SRP), NO _3_^−^ + NO_2_^−^, and NH_4_^+^ were measured using colorimetric methods with a FIA Lab FLEX system, SO_4_^2−^ via ICS-HPLC (Dionex ICS 5000+, Thermo Scientific), and total filterable Fe was measured using ICP-OES (Agilent). Suspended particulate matter (SPM) was quantified by filtering a known volume through a preweighed 47-mm 0.45 µm cellulose nitrate filter using a clean sample-rinsed filter tower. Filters were folded and stored inside a small Whirlpak bag, kept cool and in the dark. Filters were then air-dried and reweighed to calculate SPM. POC samples were collected on precombusted 47-mm GF/F filters, using a clean sample-rinsed filter tower. Filters were stored in precombusted Al foil and frozen. POC was calculated as the difference between total sediment carbon (TC) and sediment inorganic carbon (IC). TC and IC were measured on an Elementar vario PYRO cube, with samples prepared according to Hedges and Stern (14).

Dissolved methane (CH_4(aq)_) concentrations were determined as described by Hatton et al. (13). Briefly, a 1-L Nalgene bottle was filled halfway with river (sample) water and sealed with a rubber stopper. Then, the bottle was shaken for 3 min to allow CH_4(aq)_ to equilibrate with the headspace. Measurement of the headspace was conducted *in situ* using a Micro-portable Greenhouse Gas Analyser (MGGA, ABB GLA1313 Series, using Off-Axis Integrated Cavity Output Spectroscopy (OA-ICOS) technology) via a closed loop of Tygon (S3 E-3603) tubing and a hydrophobic filter (0.22 μm, PES). To evaluate local atmospheric CH_4_ corrections, atmospheric background measurements were taken above the water surface (approximately 0.5 m above the surface), and atmospheric equilibrium corresponded to 4.9 nM at ~0°C and 1 atm. Headspace CH_4_ concentrations were converted to dissolved molar concentrations using the ideal gas law (assuming atmospheric pressure) and Bunsen coefficients, following Wiesenburg and Guinasso (15).

To capture the variation in CH_4(aq)_ concentrations (Fig. S1) while maintaining comparable sample sizes across categories, we divided the sites into six CH_4_ ranges (*n* = 3–4 per group). These ranges were defined to reflect the log-scale distribution of CH_4(aq)_ concentrations: (i) Atmospheric equilibrium, sites where CH_4(aq)_ was ≤4.9 nM (*n* = 3); (ii) 4.9–20 nM (*n* = 4); (iii) 20–40 nM (*n* = 4); (iv) 40–80 nM (*n* = 4); (v) 80–490 nM (*n* = 4); and (vi) High-CH_4_ sites [sites where CH_4(aq)_ was ≥490 nM (>100 × atmospheric equilibrium); *n* = 3; see Table 1].

### Nucleic acid extraction, sequencing, and bioinformatic analysis

DNA was extracted directly from Sterivex filters using the DNeasy PowerWater Sterivex Kit (Qiagen) following the manufacturer’s protocol. DNA concentration was measured using the Invitrogen Qubit 4 Fluorometer with a Qubit 1X dsDNA HS assay kit. For the analysis of bacterial and archaeal assemblages, the V4 region of the 16S rRNA gene was PCR amplified using the primer pair 515F (Parada)–806R (Apprill), Fwd: GTGYCAGCMGCCGCGGTAA; Rev: GGACTACNVGGGTWTCTAAT (16, 17) and sequenced at SEQme s.r.o. (Dobříš, Czechia) on the Illumina NovaSeq 6000 platform using a 2 × 250-bp arrangement.

Sequence reads with an average PHRED score <30 were removed with fastp (18) v 0.23.4. Forward and reverse primers were removed with cutadapt (19) v 4.5. Then, the quality of the trimmed reads was checked using fastqc (20) v 0.12.1. Sequences were denoised, reads merged, chimeras removed, and the resulting reads grouped into amplicon sequence variants (ASVs) using the DADA2 pipeline (21). The ASVs were tentatively identified against the SILVA (22) v 138.2 database. ASVs identified as mitochondria and chloroplasts were removed from the data set. Prokaryotes present in the negative control samples that represented contamination were also removed, along with other common laboratory contaminants (such as *Ralstonia* and *Brevundimonas*), based on our internal database of contaminants and published lists (23, 24). ASVs unassigned to the phylum level were also excluded from the data set. After sequence quality control and processing, only 69 of the initial 78 samples remained. The final number of unique sites used in this study was 16 (69 total samples, Qaanaaq = 5, Upernavik = 6, Ilulissat = 10, Kangerlussuaq = 32, Nuuk = 3, Narsarsuaq = 13; see Table 1). The average number of reads per sample was 26,292, with a minimum of 7,678 and a maximum of 73,034. The final data set was rarefied to 7,678 reads and transformed to relative abundances.

To construct a phylogenetic tree for the whole data set, we performed a multiple alignment of the sequences generated by the DADA2 pipeline using the R package DECIPHER v 2.28.0 (25) and used the phangorn R package v 2.12.1 (26) to construct the tree. We first constructed a neighbor-joining tree and then fit a GTR + G + I (generalized time-reversible with gamma rate variation) maximum likelihood tree using the neighbor-joining tree as a starting point. The final tree was incorporated into a phyloseq object and used to infer the contribution of ecological processes to community assembly.

Microorganisms potentially related to CH_4_ cycling (methanogens and methylotrophs, including methanotrophs) were identified by comparing their classification with known methanogenic and methylotrophic taxa. The sequences from the potential methanogens and methylotrophs were checked by BLAST using the rRNA database (16S_ribosomal_RNA) and the Core nucleotide database (core_nt). Reference sequences, when available, were used to build a phylogenetic tree of the bacterial and archaeal methane-related taxa. Homologous sequences and query sequences were aligned in MAFFT (27) using the G-INS-i method (rest of the settings as default). Nonaligned parts of sequences were manually removed in AliView (28). The maximum likelihood phylogenetic trees were constructed with IQ-TREE v 3.0.1 (29) using automatic selection of substitution models (30) and ultrafast bootstrap analysis (31). The trees were visualized with Phylo.io (32). All ASVs that were part of the methano/methylotrophic clades in the phylogenetic tree were considered methano/methylotrophs.

### Statistical analysis

To identify the main factors shaping microbial assemblages, we examined patterns of beta diversity, environmental drivers, taxon-level responses, and community assembly processes. To visualize community dissimilarities, we created principal coordinates analysis (PCoA) ordinations based on Bray-Curtis distances of Hellinger-transformed ASV data. We tested the significance of geographical regions and sampling sites for community structure using permutational multivariate analysis of variance (PERMANOVA) with the adonis2 function in the vegan R package (33). To ensure that PERMANOVA results were not driven by unequal dispersion among groups, we tested for homogeneity of multivariate dispersions using the betadisper function in vegan.

To identify environmental predictors of community variability, we created partial distance-based redundancy analyses (dbRDA) models using environmental variables selected to represent major spatial, temporal, and biogeochemical gradients. Specifically, temporal variation was accounted for by including the day of the year (DOY) as a covariate. Spatial drivers were represented by latitude and catchment size (basin extent estimated by digital elevation model) (13, 34), and biogeochemical drivers included key solutes and particles such as SO_4_^2−^, NO_3_^−^ and total Fe (as important electron acceptors), DOC, and SRP (potentially limiting nutrients (35)), POC, and SPM (representing the particulate matrix for subglacial microbial colonization and activity), CH_4(aq)_ (a readily available carbon and energy source at the GrIS margin), and pH.

Samples with missing environmental data were excluded from multivariate analyses. The final model included 17 sampling events (corresponding to 13 sites and 47 samples in total when accounting for replicates). For DOC concentrations below the detection limit, we assigned a value of 1.5 µM (18 ppb, 0.6× the detection limit). All variables were standardized using the decostand function from the vegan package. SRP, CH_4(aq)_, SPM, DOC, Fe, NO_3_^−^, POC, and SO_4_^2−^ were log_10_-transformed before analysis. To avoid multicollinearity, we retained only variables with variance inflation factors <5. We then performed forward selection using the ordiR2step function in vegan to identify the best combination of explanatory variables. To test for changes in genus-level taxon abundance related to CH_4(aq)_ concentrations, we used ANCOM-BC (36) with log_10_-transformed CH_4(aq)_ concentrations as the predictor variable.

Finally, to infer the contribution of ecological processes to community assembly, we used the binning-based phylogenetic framework iCAMP (37) v. 1.5.12, with a bin size of 48. This employs the beta mean nearest taxon distance (βMNTD), derived from phylogenetic trees and taxonomic turnover, incorporating all bacterial ASVs in a sample, to differentiate the relative significance of homogeneous and heterogeneous selections, dispersal limitation, homogenizing dispersal, and ecological drift. For this analysis, replicates from the same sampling event were merged using the merge_samples() function from the phyloseq R package (38) and then rarefied to 50,225 reads per sample (the lowest number of reads after sample merging) prior to iCAMP analyses. Data visualization was conducted using the R packages ggplot2 version 3.5.2 (39) and microViz version 0.10.10 (40).

## RESULTS AND DISCUSSION

### Microbial assemblage composition

The microbial assemblages exhibited a taxonomic composition consistent with previous glacial ecosystem studies, dominated by the phylum Pseudomonadota, followed by Bacteroidota and Actinomycetota (41, 42). A total of 6,837 ASVs were identified in the rarefied data set, with the five most abundant ASVs (from highest to lowest) belonging to the genera *Rhodoferax*, *Polaromonas*, *Methylotenera*, *Rhodoferax*, and *Crenothrix*, respectively. This assemblage structure is comparable to those reported in previous studies of Greenland glacial meltwater (3, 5, 6) and sediments (7, 43). Archaeal ASVs accounted for 2.5% of the total number of ASVs in the data set, representing ~0.23% of the total reads.

The widespread dominance of *Polaromonas* and *Rhodoferax* ASVs among the samples aligns with previous findings that identify these genera as typical constituents of the cryospheric microbiome (42). Both are known for their metabolic adaptability, with *Rhodoferax* being able to switch between anaerobic and aerobic lifestyles (44), and *Polaromonas* being a generalist able to utilize a variety of carbon substrates (45). These strategies may be useful in the subglacial environment, where variable redox conditions and OM sources are present (46, 47).

### Assemblage patterns and environmental drivers

**Fig 2:**
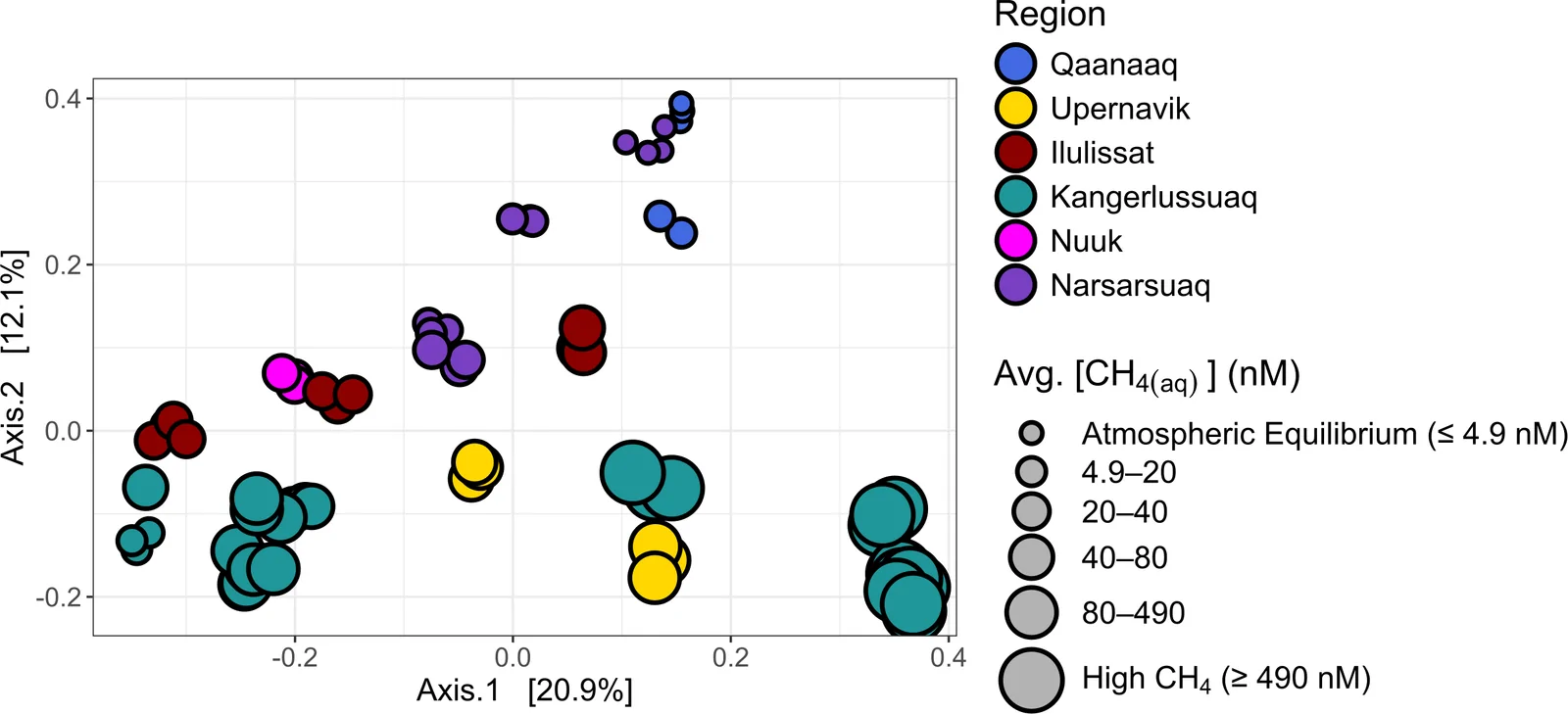
Principal coordinates analysis (PCoA) showing differences between sites as a function of their geographic region and CH_4(aq)_ concentrations.

PCoA explained 33% of the total variance across the first two components (Fig. 2). PERMANOVA showed that regions significantly explained some of the community dissimilarity (*R*^2^ = 0.281, *F* = 4.929, *P* = 0.001). However, the dispersions of sites within the groups were also significantly different from each other (*F* = 11.144, *P* = 0.001). Notably, sites with very high CH_4(aq)_ concentrations (SW4, SW5-a,c,d) clustered together and were clearly separated from sites with lower CH_4(aq)_ concentrations.

**Fig 3:**
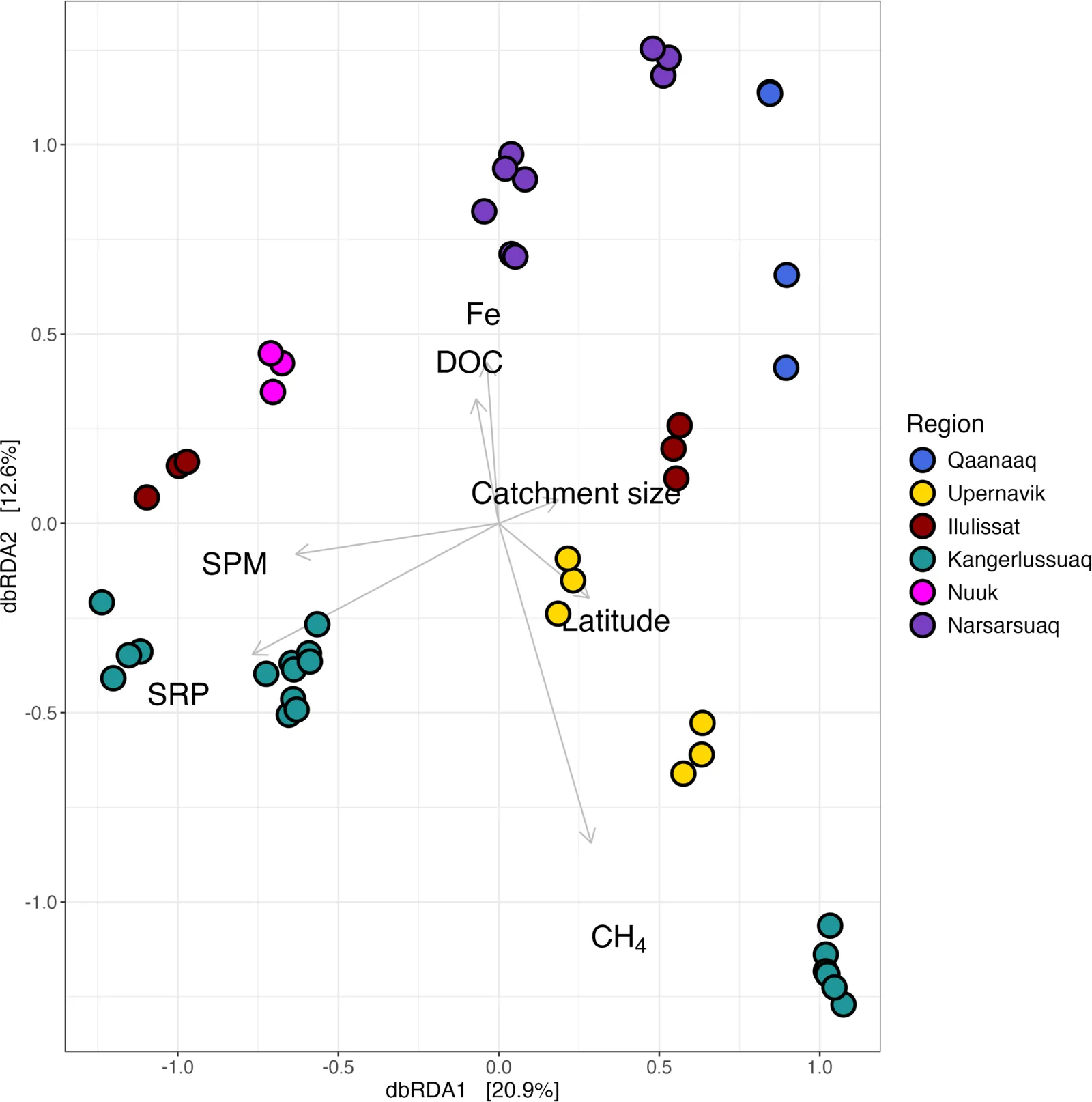
Partial distance-based redundancy analysis (dbRDA) illustrating the most parsimonious set of variables accounting for variation in assemblage structure. Sites lacking environmental data were omitted from the analysis (see Table 1; Table S1).

Both carbon acquisition strategies and physical habitat structure play important roles in shaping subglacial microbial assemblages, as indicated by partial distance-based redundancy analyses (dbRDA) using Bray-Curtis distances on Hellinger-transformed data. The most parsimonious model included CH_4(aq)_ as the strongest factor (*F* = 8.766, *P* = 0.001), with contributions also from soluble reactive phosphorus (SRP) (*F* = 4.619, *P* = 0.001), suspended particulate matter (SPM (*F* = 4.133, *P* = 0.001), latitude (*F* = 6.435, *P* = 0.001), DOC (*F* = 3.095, *P* = 0.003), catchment size (*F* = 3.932, *P* = 0.003), and Fe (*F* = 2.983, *P* = 0.003). The first dbRDA axis explained 20.9% of the variance and was primarily associated with SPM and SRP, while the second axis explained 12.6% and was dominated by CH_4(aq)_, with additional contributions from DOC, latitude, and Fe (Fig. 3). The full model explained 43% of community variation (adj. *R*^2^ = 0.436; *R*^2^ = 0.505) and remained significant after accounting for sampling day (DOY; 6.97% of variance; ANOVA, *F* = 6.46, *P* = 0.001).

CH_4_ emerged as the strongest individual predictor (Fig. 3), highlighting the importance of methanogenesis and methylotrophy in these environments. The loading of SPM on the first component likely reflects its export from subglacial habitats, where sediment particles can act as carriers of mineral-associated microbes and associated nutrients. This pattern likely represents the downstream transport of sediment-bound nutrients originating beneath the ice, a process widely documented in glacial systems (48, 49). DOC structured variability along the second component, likely reflecting differences in organic carbon availability that influence heterotrophic activity in the subglacial environment.

SRP concentration was also an important factor in structuring the exported assemblages. This is consistent with recent evidence of P limitation in glacier-fed streams and their benthic microbiomes (35), despite potentially high glacial bedrock-derived P inputs (47, 50). Iron also emerged as an important predictor of community composition, perhaps reflecting the ubiquity of Fe-cycling taxa such as *Rhodoferax*, alongside iron reducers (e.g. *Ferruginibacter*) and iron oxidizers (e.g., *Thiobacillus*, *Sideroxydans*). Although this is suggestive of a role for Fe cycling in structuring microbial communities at the GrIS margins, its interpretation is limited because our measurements represent total filterable Fe and provide no information on Fe valence.

### Microbial assemblage variation along a CH_4_ concentration gradient

CH_4_-related taxa comprised 83 ASVs, including both methanogens and methylotrophs (Fig. S2 to S4). Since CH_4(aq)_ concentration emerged as the strongest individual predictor of the microbial assemblage structure, we further examined changes in CH_4_-related taxa in their response to the CH_4(aq)_ gradient.

**Fig 4:**
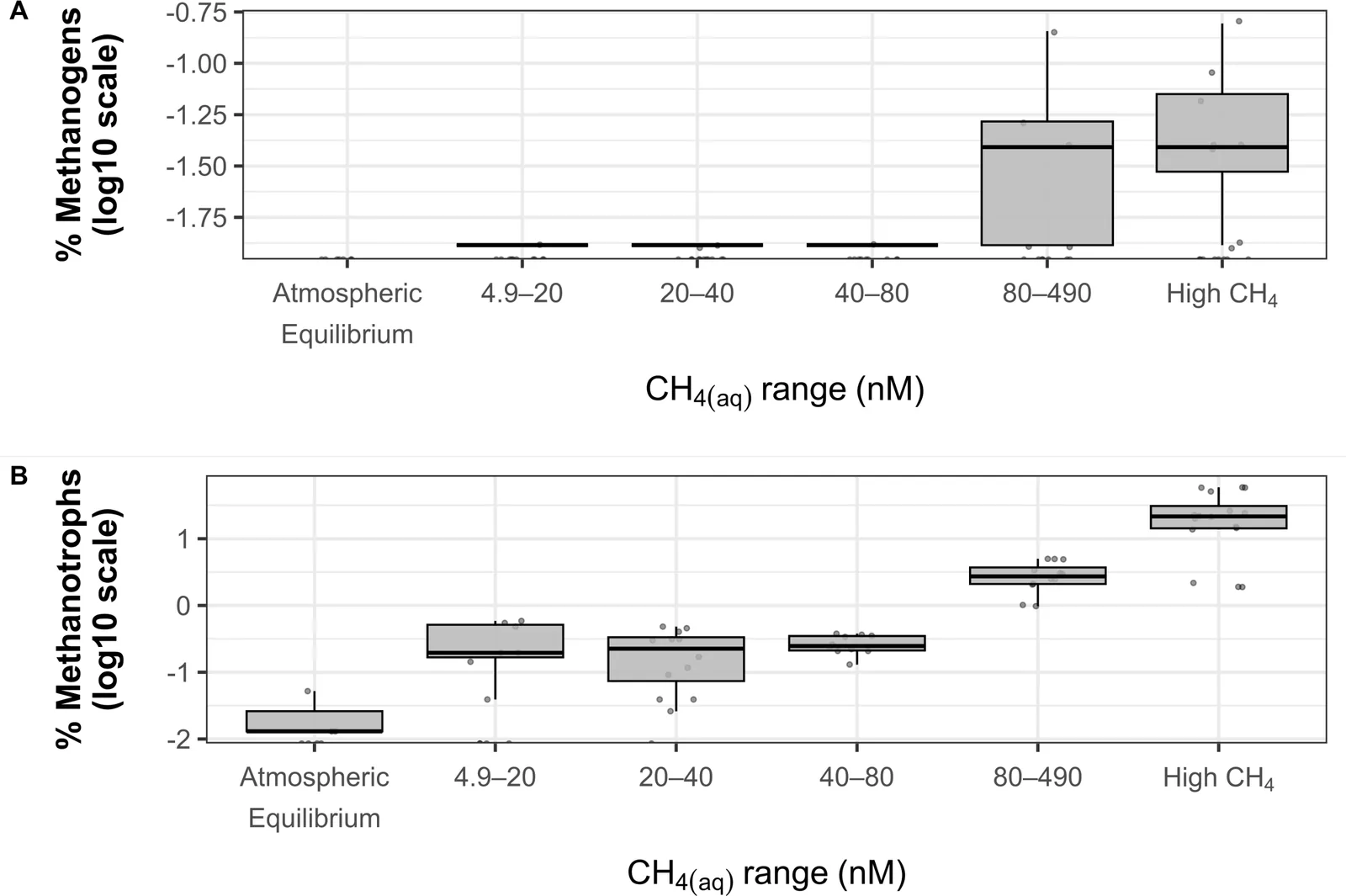
Relative abundance of methanogens and methanotrophs across the different CH_4(aq)_ ranges. (**A**) Relative abundance of methanogens (in log10 scale). (**B**) Relative abundance of methanotrophs (in log10 scale).

The relative abundance of methanogens was very low (0.01%–0.15%; Fig. 4), with the most abundant ASV (ASV 1147, *Methanoregula* sp., Methanomicrobiales) reaching only 0.004%. Additional methanogenic taxa affiliated with the genera *Methanobacterium*, *Methanosphaerula*, *Methanothrix*, and the families Rice Cluster II and Methanomassiliicoccaceae were detected. Methanogens were present at their highest relative abundances at all high-CH_4_ sites and at least one site per intermediate CH_4(aq)_ range (4.9–490 nM) but absent from all atmospheric equilibrium sites. Low methanogen abundances are consistent with previous studies at similar sites (3, 9), likely reflecting methanogenesis occurring in deeper sediment layers or isolated subglacial zones where cells are retained in sediments while the CH_4_ produced is evacuated into subglacial drainage pathways and flushed downstream in meltwater. Despite low abundances, a phylogenetically diverse set of methanogenic archaea spanning four orders was detected (Fig. S2). While CH_4(aq)_ isotopic signatures from the glacier outlets of the Kangerlussuaq region (Isunnguata Sermia, Leverett Glacier) suggest that acetoclastic methanogenesis dominates CH_4_ production (9, 10, 13), the assemblages in this study were dominated by hydrogenotrophic methanogens (*Methanoregula*, *Methanobacterium*, *Methanosphaerula*, Rice Cluster II). Lamarche-Gagnon et al. (9) observed the same pattern, noting the difficulty of inferring methanogenic contributions to the CH_4(aq)_ pool from abundance data only.

In contrast to methanogens, methylotrophic taxa were abundant and strongly associated with elevated CH_4(aq)_ concentrations (Fig. 4). Six methylotrophic ASVs affiliated with the genera *Methylotenera*, *Methylobacter*, and *Crenothrix* reached relative abundances of 1.1%–4.3% and together comprised 25%–76% of the total assemblage at most high-CH_4_ sites. These taxa are commonly enriched in cryospheric environments with high CH_4_ fluxes, where they act as an important biological sink for CH_4_ (51–53).

The most abundant methylotrophs (i.e., *Methylotenera*, *Crenothrix*, and *Methylobacter*) occupy complementary niches in the CH_4_ cycle. *Methylotenera* oxidizes methanol, formate, and other C1 compounds but not CH_4_ directly, often coexisting with methanotrophs such as *Methylobacter* that provide methanol from CH_4_ oxidation (54, 55). *Methylotenera*’s ubiquity, even at atmospheric CH_4_ sites, where it likely utilizes C1 by-products from organic matter degradation (3), suggests it is also a core component of the cryospheric microbiome. Methanotrophic communities were dominated by Type I methanotrophs (family Methylomonadaceae; 31 ASVs, 8.9% of reads), primarily *Crenothrix* and *Methylobacter*. Type II (Alphaproteobacteria) and Type III (Verrucomicrobia) methanotrophs were rare (0.0002–0.01 % of reads). Type I dominance is consistent with their adaptation to cold, CH_4_-rich, and O_2_-limited conditions typical of subglacial environments (3, 55), where they likely outcompete Type II taxa, as observed in other high-CH_4_ Arctic systems (52).

**Fig 5:**
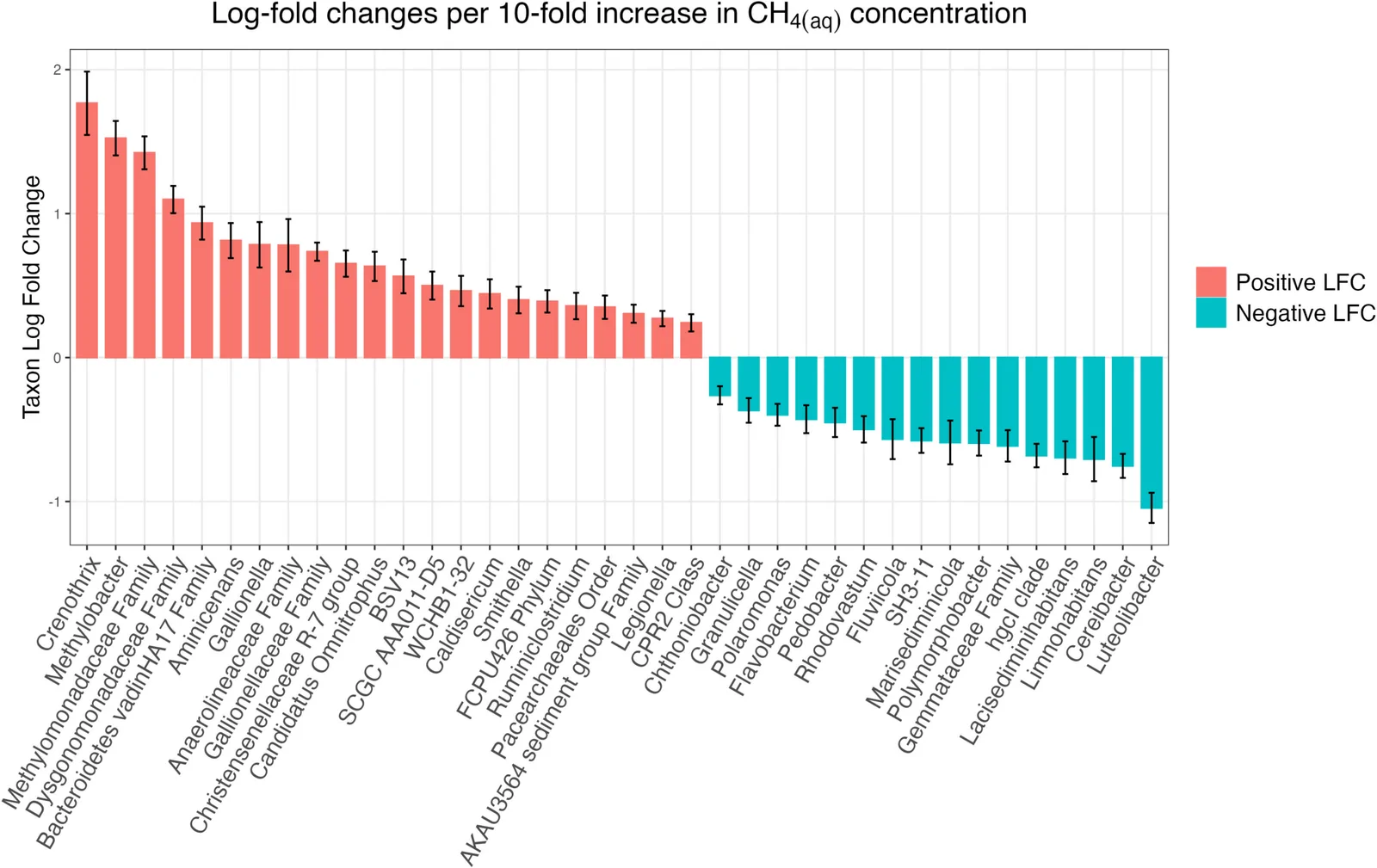
Differential abundance analysis showing the taxonomic differences with increasing CH_4(aq)_ concentrations. Positive log fold-change (LFC) values indicate an increase in taxa abundance with increasing CH_4(aq)_ concentrations; negative log fold-change values indicate a decrease in abundance with increasing CH_4(aq)_ concentrations. CH_4(aq)_ concentrations were log-transformed before the analysis, and taxon log fold changes correspond to a 10-fold increase in CH_4(aq)_ concentration.

Differential abundance analysis showed 38 taxa across 13 phyla whose relative abundances significantly changed with increasing CH_4(aq)_ concentrations (Fig. 5). Of these, 22 taxa increased and 16 decreased in their relative abundances with rising CH_4(aq)_. The strongest positive responses were observed for *Crenothrix*, *Methylobacter*, and Methylomonadaceae, followed by Dysgonomonadaceae, Bacteroidetes vadinHA17, *Aminicenans*, *Gallionella*, and Anaerolineaceae. In contrast, taxa such as *Luteolibacter*, *Cereibacter*, *Limnohabitans*, *Lacisediminihabitans*, and the hgcl clade showed strong negative associations with CH_4(aq)_.

The enrichment of *Crenothrix*, *Methylobacter*, and Methylomonadaceae at high CH_4(aq)_ concentrations supports their roles as active CH_4_ consumers and microbial sinks, particularly at high-CH_4_ sites, where they comprise up to 59% of the assemblage. Notably, the next most enriched taxa were Bacteroidota members Dysgonomonadaceae and Bacteroidetes vadinHA17. Given that subglacial organic matter can derive from terrestrial vascular plants (47), the degradation of lignocellulose into short-chain fatty acids (e.g., acetate) by Dysgonomonadaceae (56) likely provides essential precursors for methanogenesis. The enrichment of taxa that can degrade complex polysaccharides (*Aminicenans*, *Ruminiclostridium*) (57, 58) suggests active breakdown of OM under subglacial conditions. Additional enrichment of taxa that release acetate, formate, and/or H_2_ as by-products (Christensenellaceae R-7 group, Anaerolineaceae, Candidatus *Omnitrophus*, *Caldisericum*, Pacearchaeales) (59–63) highlights the production of key intermediates for methanogenesis. The presence of taxa that live in syntrophic association with H_2_- or formate-utilizing microorganisms (*Smithella*) (64) further suggests that complex OM degradation and CH_4_ production are tightly coupled, indicating an active microbial carbon-processing network beneath the ice.

### Ecological processes driving community assembly

**Fig 6:**
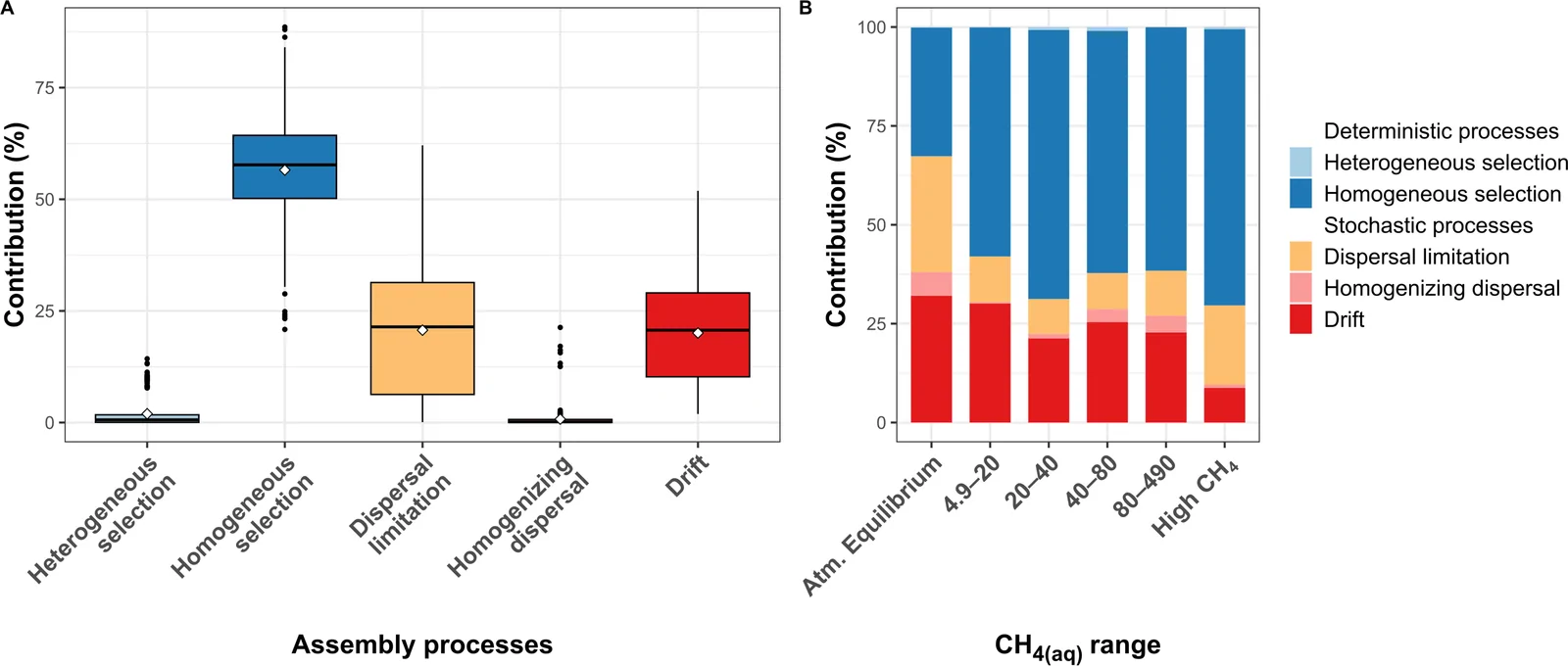
Community assembly processes inferred by phylogenetic overdispersion (iCAMP) analyses. (**A**) Overall contribution of processes to community assembly for the whole data set. (**B**) Average contribution of processes to community assembly in each CH_4(aq)_ range.

Using phylogenetic overdispersion to infer the dominant mechanisms structuring sampled meltwater assemblages, we found that homogeneous selection (selection acting against community differentiation) was the most common assembly process (56.5% of community pairs; Fig. 6), followed by dispersal limitation (20.7%), drift (20%), heterogeneous selection (selection promoting the differentiation of communities; 2%), and homogenizing dispersal (dispersal rates promoting community similarity; 0.8%). The predominance of homogeneous selection indicates that consistent environmental controls are acting to select for similar microbial assemblages across multiple sites and is consistent with previous studies on benthic biofilms in glacier-fed streams (65–67). Notably, in our study, deterministic processes were significantly more important in explaining the assembly of high-CH_4_ sites and sites with CH_4(aq)_ ranges of 20–40 nM compared to sites with atmospheric CH_4(aq)_ concentrations (Kruskal-Wallis rank sum test, followed by Dunn test, *P* = 0.015; Fig. 6). This suggests that CH_4(aq)_ availability imposes strong and uniform selection pressures on the microbial communities.

At high-CH_4_ sites, the dominance of homogeneous selection was driven by a small number of phylogenetic bins containing highly abundant and functionally relevant taxa. Bin 9, dominated by *Methylotenera*, and bin 11, which includes *Rhodoferax* and *Polaromonas* (Extended Data Table 1), are commonly reported from glacial ecosystems (42) and are known for their capacity to thrive under cold, low-nutrient conditions. Bin 13, composed of the methanotrophs *Crenothrix*, *Methylobacter*, and *Methylomonadaceae*, accounted for over 23% of the homogeneous selection signal. This suggests that CH_4_-consuming taxa are strongly selected for under high-CH_4_ conditions, leading to convergence in community structure. These bins also contain some of the most abundant and widespread taxa in the data set, which supports the notion that core microbiome members can disproportionately shape deterministic assembly patterns and is consistent with findings in other cryospheric systems (65, 67).

In contrast, the sites where CH_4(aq)_ was around or below atmospheric equilibrium were characterized by a significantly weaker influence of homogeneous selection and a proportionally greater role for stochasticity. This is consistent with ecological theory, where stochastic processes become more dominant in the absence of strong environmental filtering (68, 69). Consequently, communities at the low-CH_4_ sites are less dominated by specialized consortia and are more susceptible to stochastic events (70), resulting in more divergent, stochastic assemblages, mostly composed of generalist aerobes, as identified by our differential abundance analysis.

At the intermediate CH_4(aq)_ concentration sites, homogeneous selection remained a dominant process, but its phylogenetic drivers shifted away from the specialized methanotrophic consortium. Here, selection was primarily directed by bins associated with versatile metabolic strategies and redox interfaces. For instance, bin 11 (containing *Polaromonas* and *Rhodoferax*) and bin 12 (including the iron-oxidizers *Sideroxydans* and *Gallionella*) were prominent, especially in the ranges of 4.9–20 nM and 40–490 nM. This suggests that at moderate CH_4(aq)_ levels, environmental filtering is not controlled by CH_4(aq)_ concentrations alone and depends more on the broader redox environment that accompanies CH_4_ production and oxidation, including iron cycling, oxygen availability, redox potential, and the availability of alternative electron donors and acceptors. Oxygen availability is also likely to play a major role in structuring these communities, particularly given its direct regulation of CH_4_ oxidation. Although dissolved oxygen concentrations measured in the emerging meltwaters were consistently near saturation, these values likely reflect rapid atmospheric equilibration during turbulent transport and emergence of low-temperature meltwater rather than *in situ* redox conditions within the subglacial drainage system. Oxygen availability beneath the ice is therefore expected to be considerably more heterogeneous and may represent an additional deterministic driver of microbial community assembly that was not captured by our field measurements.

The observation that homogeneous selection was highest in both high-CH_4_ sites and the 20–40 nM range but did not increase linearly with CH_4(aq)_ concentration suggests a saturation or threshold effect in how CH_4(aq)_ concentration structures community assembly. At atmospheric equilibrium CH_4(aq)_ concentrations, environmental filtering is weak and stochastic processes likely dominate. As CH_4(aq)_ concentration increases, it starts exerting selective pressure (and/or implies an environment where strong selective pressure can occur due to strong redox gradients). Further increase in CH_4(aq)_ concentrations to high-CH_4_ site levels likely maintains this deterministic effect but does not amplify it further, as the functional niche for methanotrophs and their syntrophic partners is already saturated. This pattern mirrors ecological saturation dynamics observed in other systems where resource availability above a critical threshold selects for specialized functional guilds, but additional resource inputs do not proportionally increase deterministic assembly once the niche is fully occupied (69, 71, 72).

Finally, the stronger influence of homogeneous selection observed at high-CH_4_ sites suggests that CH_4_ works as a specialized ecological filter rather than simply representing an additional carbon source. It can only be directly oxidized by methanotrophs, and elevated CH_4_ concentrations can therefore selectively favor microorganisms that possess CH_4_ oxidation pathways. At the same time, the metabolic activity of these taxa can subsequently restructure the wider community through carbon cross-feeding (for example, providing methanol for *Methylotenera*), localized oxygen depletion, and interactions with other biogeochemical processes. As a result, CH_4_ availability can promote deterministic assembly not only by directly selecting CH_4_ oxidizers but also by indirectly shaping the ecological niches available to non-methanotrophic community members. Ultimately, CH_4_ can be best interpreted as a proxy for, and component of, the underlying redox gradient that structures microbial community assembly.

### Conclusion

Microbial assemblages exported from terrestrial outlets of the GrIS are shaped primarily by homogeneous selection, with CH_4(aq)_ concentrations acting as a key environmental filter that structures community composition and function. CH_4(aq)_ concentration does not act as a linear selector of microbial communities but rather defines assembly regimes. At one extreme, high-CH_4_ sites are deterministic engines, where homogeneous selection enforces the assembly of a functionally coherent consortium centered on CH_4_ cycling; at the other end of the spectrum, low, atmospheric equilibrium CH_4(aq)_ sites show more stochastic processes, where drift and dispersal create more variable communities of generalists. The presence of syntrophic taxa and complex polysaccharide degraders in association with increasing CH_4_ concentrations further suggests that internal microbial interactions reinforce CH_4_ cycling by linking methanogenesis and methanotrophy with upstream organic matter degradation processes; however, further research is necessary to confirm functional interactions.

## Data Availability

The raw 16S rRNA gene sequence data are available from the NCBI Sequence Read Archive under BioProject PRJNA1289195. Metadata and processed sequences used for the statistical analyses in this study have been deposited in the Zenodo repository under accession number 22085960. The codes used in the study are available on the GitHub repository at https://github.com/CryoEco/MARCH4G-microbial.

## SUPPLEMENTAL MATERIAL

Supplemental material aem.00875-26-s0001.docx Table S1; Fig. S1 to S4.

ASM does not own the copyrights to Supplemental Material that may be linked to, or accessed through, an article. The authors have granted ASM a non-exclusive, world-wide license to publish the Supplemental Material files. Please contact the corresponding author directly for reuse.
