# Supplementary material for: Methane is an important selection factor in microbial community assembly along the Greenland Ice Sheet margin
Source: Appl Environ Microbiol. 2026 Sep 15;92(10):e00875-26. doi: 10.1128/aem.00875-26 (PMC13689279; doi:10.1128/aem.00875-26)
Supplement: Supplementary file 1 [file aem.00875-26-s0001.docx]

Supplementary information for

**Methane is an important selection factor in microbial community assembly along the Greenland Ice Sheet margin**

Lia C.P. Wentzel^1*^, Petra Klímová^1^, Tyler J. Kohler^1^, Jade E. Hatton^1,3^, Anna Stehrer-Polášková^1^, Jakub D. Žárský^1^, Jakub Trubač^2^, Philip A. Píka^1,4^, Jon R. Hawkings^5,6^, Eva L. Doting^6,7^, Jack G. Murphy^6^ and Marek Stibal^1^

^1^Department of Ecology and ^2^Institute of Geochemistry, Mineralogy and Mineral Resources, Faculty of Science, Charles University, Prague, Czechia; ^3^UK Centre for Ecology & Hydrology, Environment Centre Wales, Bangor, UK; ^4^Geosciences, Environment and Society Department, Université Libre de Bruxelles, Brussels, Belgium; ^5^iC3—Centre for Ice, Cryosphere, Carbon and Climate, Institute for Geosciences, UiT - The Arctic University of Norway, Tromsø, Norway; ^6^Department of Earth and Environmental Science, University of Pennsylvania, Philadelphia, PA, USA; ^7^Department of Arctic Ecology, Norwegian Institute for Nature Research, Tromsø, Norway

***Corresponding Author**: Lia Wentzel

Email: costapil@natur.cuni.cz

ORCIDid: <https://orcid.org/0000-0001-9018-8569>

Table S1. Physical and chemical characteristics of the collected samples. Samples marked with * were not included in any analyses due to an insufficient number of reads and/or low quality. SRP, DOC, Total filterable Fe, SO_4_^2-^ and NO_3_^-^ are in ppb; POC and SPM are in g L^-1^. When more than one value is reported for a sample, these correspond to replicate measurements.

| Sample | Sample name in SRA | Stream type | Catchment size (km^2^) | Vol. water filtered (mL) | Avg. Measured CH_4(aq)_ (nM) | SRP | DOC | Total filt. Fe | SO_4_^2-^ | POC | SPM | NO_3_^-^ | pH |
| --- | --- | --- | --- | --- | --- | --- | --- | --- | --- | --- | --- | --- | --- |
| NW1 | QA-02 | Lateral glacial stream | 96.8 | 3 x 160 | 0.83 | 2.07 | 82.98 | 38 | 705 | 0.00006  0.00013  - | 0.15 | 22.46 | 6.86 |
| NW2 | QA-01 | Lateral glacial stream | 6.9 | 3 x 160 | 13.9 | 2.17 | 502.36 | 29.5 | 759.05 | 0.00282 | 0.25 | 4.47 | 6.97 |
| NW3 | UP-02 | Subglacial portal | 517 | 3 x 200 | 179.7 | 1.93 | 255.88 | 90 | 796.1 | 0.00026  0.00026  0.00029 | 1.07 | 15.05 | 7.1 |
| NW4 | UP-01 | Proglacial spring | 3490 | 1 x 250,  1 x 200,  1 x 160 | 62.84 | 4.61 | 250.21 | 147 | 2041.2 | 0.0123 | 2.72 | 22.96 | 7.6 |
| CW4-a | IL-02-a | Subglacial portal | 6.1 | 3 x 240,  1 x 120 | 38.17 | 5.22 | <LoD | 346 | 1845 | 0.00018  0.00014  0.00028  - | 0.6 | 17.65 | 7.12 |
| CW4-b | IL-02-b | Lateral glacial stream | 6.1 | 3 x 180 | 34.43 | 1.5 | 223.07 | 97 | 1368.4 | NA | NA | NA | 7.6 |
| CW5 | IL-01 | Lateral glacial stream | 703000 | 3 x 200 | 75.84 | 2.71 | 159.96 | 83 | 367.7 | 0.00013 | 0.15 | 4.47 | 7.45 |
| SW3-f | KA-09 | Subglacial spring | 14100 | 1 x 240, 2 x 216 | 65.95 | 7.22 | 673.51 | 89 | 5749.9 | 0.00022 | 0.4 | 15.42 | 6.88 |
| SW3-a* | KA-03-a | Proglacial spring | 14100 | 3 x 240 | 9.68 | 21.98 | 172.96 | 825 | 4491.1 | 0.00049 | 2.1 | 24.61 | 7.7 |
| SW3-b | KA-03-b | Proglacial spring | 14100 | 1 x 200,  1 x 225,  1 x 125 | 136.04 | 5.41 | 281.23 | 13 | 1910.9 | 0.00052 | 2.75 | 23.03 | 9.3 |
| SW3-c | KA-03-c | Proglacial spring | 14100 | 3 x 150 | 95.36 | 6.14 | 192.98 | 18 | 2034.1 | 0.0009 | 0.79 | 21.96 | NA |
| SW3-d | KA-03-d | Proglacial spring | 14100 | 3 x 200 | 6.33 | 10.33 | 305.47 | 14 | 951.5 | 0.00034  0.00024  0.00022 | 1.4 | 14.59 | 8.3 |
| SW4 | KA-02 | Proglacial spring | 1 | 3 x 240,  1 x 160 | 49613.26 | NA | 664.18 | 73 | 366.9 | 0.00076 | 0.04 | NA | 6.89 |
| SW5-a | KA-01-a | Lateral glacial stream | 6.2 | 3 x 240 | 490 | 2.36 | 47.38 | 17 | 265.9 | 0.00067  0.00136  0.00167 | 0.3 | 5.07 | 7.3 |
| SW5-c | KA-01-c | Lateral glacial stream | 6.2 | 3 x 200 | 1331.7 | 2.43 | 61.51 | 98 | 378.05 | 0.00067 | 0.16 | 5.77 | 6.93 |
| SW5-d | KA-01-d | Lateral glacial stream | 6.2 | 3 x 200 | 2238.37 | 0.83 | 1008.67 | 42 | 459.6 | NA | 0.25 | 4.99 | 6.5 |
| SW6-a | KA-04-a | Subglacial portal | 782 | 3 x 240 | 60.52 | 13.02 | 73.71 | 93 | 5653.1 | 0.00046 | 0.57 | 23.36 | 9.5 |
| SW6-b | KA-04-b | Subglacial portal | 782 | 3 x 120 | 291.56 | 10.35 | 1021.21 | 188 | 1740.3 | NA | 1.59 | NA | 8.16 |
| SW7 | KA-05-a | Lateral glacial stream | 7 | 3 x 200 | 14026.96 | 2.45 | 819.73 | 121 | 363.6 | NA | 0.88 | NA | 6.1 |
| SW12 | NU-01 | Proglacial spring | 7674.8 | 3 x 200 | 21.53 | 5.41 | 175.37 | 230 | 3205 | 0.00045  0.00035  0.00013 | 2.6 | 17.66 | 7.2 |
| SW12-b* | NU-02 | Proglacial spring | 7674.8 | 3 x 200 | 17.88 | 5.41 | 124.68 | 86 | <200 | 0.00002 | 0.75 | 0.79 | 7.8 |
| SW13 | NA-01 | Subglacial portal | 19.6 | 3 x 200 | 15.5 | 1.63 | 415.43 | 87 | 3370.9 | 0.00002 | 0.34 | 6.17 | 7.4 |
| SW14-a | NA-02 | Subglacial portal | 13.5 | 3 x 200 | 11.51 | 3.22 | 435.15 | 259 | 312.2 | 0.00036 | 0.43 | 6.98 | NA |
| SW14-b | NA-04 | Subglacial portal | 13.5 | 3 x 200 | 19.46 | 0.63 | 270.07 | 140 | 384.1 | 0.0006 | 1.05 | 5.38 | NA |
| SW15 | NA-03 | Lateral glacial stream | 34.4 | 2 x 400,  1 x 200 | 2.98 | 0.95 | 205.17 | 18 | 561.3 | NA | NA | 5.58 | 7.5 |
| SW15-b | NA-05 | Lateral glacial stream | 34.4 | 1 x 200 | 0.99 | 3.32 | NA | NA | NA | NA | NA | 7.42 | NA |

Figure S1. CH_4(aq)_ concentrations measured across sampling sites. Red dashed lines represent the thresholds for the CH_4(aq)_ ranges.

Figure S2. Maximum likelihood tree of CH_4_-related archaea detected in the assemblages (in bold), rooted with the sequences of *Pyrococcus furiosus* and *Thermococcus kodakarensis*.

Figure S3. Maximum likelihood tree of CH_4_-related bacteria (methylo- and methanotrophs) from the phyla Pseudomonadota and Methylomirabilota detected in the assemblages (in bold). The tree was rooted with sequences of *Acidobacterium capsulatum* and *Acidipila rosea*.

Figure S4. Maximum likelihood tree of methanotrophs from the phylum Verrucomicrobiota detected in the assemblages (in bold). The tree was rooted with sequences of *Rhodopirellula baltica* and *Rubripirellula lacrimiformis*.
